# TS: a powerful truncated test to detect novel disease associated genes using publicly available gWAS summary data

**DOI:** 10.1186/s12859-020-3511-0

**Published:** 2020-05-04

**Authors:** Jianjun Zhang, Xuan Guo, Samantha Gonzales, Jingjing Yang, Xuexia Wang

**Affiliations:** 1 Department of Mathematics, University of North Texas, 1155 Union Circle #311430, Denton, 76203 TX USA; 2 Department of Computer Science and Engineering, University of North Texas, Discovery Park 3940 N. Elm, Denton, 76203 TX USA; 3 Center for Computational and Quantitative Genetics, Department of Human Genetics School of Medicine, Emory University, Whitehead Biomedical Research Building, Suite 305K, Atlanta, 30322 GA USA

**Keywords:** Genome-wide association studies (GWAS), Quadratic test methods, Burden tests, Truncated statistic method

## Abstract

**Background:**

In the last decade, a large number of common variants underlying complex diseases have been identified through genome-wide association studies (GWASs). Summary data of the GWASs are freely and publicly available. The summary data is usually obtained through single marker analysis. Gene-based analysis offers a useful alternative and complement to single marker analysis. Results from gene level association tests can be more readily integrated with downstream functional and pathogenic investigations. Most existing gene-based methods fall into two categories: burden tests and quadratic tests. Burden tests are usually powerful when the directions of effects of causal variants are the same. However, they may suffer loss of statistical power when different directions of effects exist at the causal variants. The power of quadratic tests is not affected by the directions of effects but could be less powerful due to issues such as the large number of degree of freedoms. These drawbacks of existing gene based methods motivated us to develop a new powerful method to identify disease associated genes using existing GWAS summary data.

**Methods and Results:**

In this paper, we propose a new truncated statistic method (TS) by utilizing a truncated method to find the genes that have a true contribution to the genetic association. Extensive simulation studies demonstrate that our proposed test outperforms other comparable tests. We applied TS and other comparable methods to the schizophrenia GWAS data and type 2 diabetes (T2D) GWAS meta-analysis summary data. TS identified more disease associated genes than comparable methods. Many of the significant genes identified by TS may have important mechanisms relevant to the associated traits. TS is implemented in C program TS, which is freely and publicly available online.

**Conclusions:**

The proposed truncated statistic outperforms existing methods. It can be employed to detect novel traits associated genes using GWAS summary data.

## Background

Even though genome-wide association studies (GWASs) have been remarkably successful in identifying a large number of genetic variants associated with complex traits and diseases, these identified variants can only explain a small to modest fraction of heritability [1]. Larger sample sizes and more powerful statistical tests are needed to boost power to identify novel genetic variants, especially for weakly associated variants with small effect sizes or low frequency variants. Due to various reasons, it is often difficult for researchers to obtain access to individual level data, and thus difficult to obtain a sufficient sample size to obtain reliable results. The increase in public availability of genome-wide association study (GWAS) summary statistics, e.g. minor allele frequency (MAF), estimated effect size, odds ratio, or p-values, for individual single nucleotide polymorphisms (SNPs) motivated us to develop novel powerful methods for analyzing GWAS summary data. Methods based on summary statistics can also be viewed as a complementary approach to the traditional single variant single trait association test.

Most popular gene based association tests (grouping SNPs together into a SNP set (e.g. a gene or surrounding of a gene) to test the joint effects of SNPs in the SNP set [2]) can often be viewed as a combination of summary statistics (e.g. Z statistics or p-values generated from GWAS). For example, the weighted sum statistic (WSS) [3] is a type of burden test statistic, which is used to jointly analyze a group of genetic variants in order to test for association in a considered region. The WSS can be viewed as a weighted sum of the summary statistic, where the summary statistic is the Z statistic. The sequence kernel association test (SKAT) [4] and the sum of squared score test (SSU) [5] are proposed to test for association between genetic variants and a single trait. Both tests can be viewed as the weighted sum of summary statistics, where the summary statistic is a score test. The SKAT-O statistic [6] is a linear combination of SKAT and the burden test. When a tuning parameter searching in a certain range, the SKAT-O can reach the maximized value of power. The burden test and SKAT can be considered as special cases of SKAT-O. The SKAT-O statistic can also be rewritten as a special combination of Z statistics [7]. In addition to the aforementioned gene based association tests, there are several other p-value based methods which are not based on Z statistics, such as the minimum p-value, a general gene-based p-value adaptive combination approach (GPA) [8] or the gene-based association test, which uses extended Simes procedure (GATES) [9].

Most of the existing gene-based methods can be viewed as either burden test methods or quadratic test methods. Burden test methods are usually powerful when the direction of effects of genetic variants in the considered region are the same. Quadratic test methods usually have reasonable power given a wide range of alternative hypotheses. Specifically, burden tests collapse the variants in a genomic region into a single burden variable by using weighted combination of variants, and then test the association between the trait and the single burden variable. Quadratic tests refer to tests with statistics of quadratic forms of the score vector. However, burden tests can suffer loss of statistical power when different directions of effects of causal variants exist, while quadratic tests could be less powerful due to other issues such as the large number of degree of freedoms. Thus, we aim to develop a new powerful method for further analyzing GWAS summary data. In the “Method” section of this paper, we propose a truncated statistic method (TS) to find approximate contributions of trait associated genes. As our method is based on the quadratic test method and uses a truncated statistic to find the most likely contributions of genetic variants, TS can overcome the shortcomings of both quadratic and burden tests.

To evaluate the performance of the proposed method, we conducted extensive simulation studies and real data analysis. We compared our method TS with five existing gene-based methods: a gene-based association test that uses an extended Simes procedure (GATES) [9], an adaptive sum of powered score tests (aSPU) [10], and three methods proposed by [11]: sum test (ST), squared sum test (S2T), and the adaptive test (AT). All methods are designed for single trait association studies. GATES adopts an extended Simes procedure to correct multiple testing issues while calculating the p-value quickly based on SNP summary statistics. The aSPU method estimates and selects the most powerful test among a class of so-called, sum of powered score (SPU) tests. ST is a type of burden test statistic [3], S2T is a type of SKAT statistic [4], and AT is equivalent to the SKAT-O statistic [6]. Simulation results demonstrate that our proposed method TS outperforms the five comparable methods. Results of our application to the schizophrenia GWAS summary data obtained from the Psychiatric Genomics Consortium (PGC), and fasting glucose GWAS meta-analysis summary data obtained from the UK Biobank component of the European DIAMANTE study, also indicate that our method performs better than existing methods.

## Results

### Simulations

We conducted extensive simulation studies to evaluate the performance of TS. We compared the type I error rates and power of TS with the five existing methods by following the simulation setting in [12]. Kwak et al. [10] have shown that the performance using any reference data from the same ancestry in estimating linkage disequilibrium (LD) among SNPs is mostly satisfactory with an estimated inflation factor close to 1. Therefore, the LD between SNPs in the simulation studies is estimated using the 1000 Genomes project [13].

#### Type I error

To evaluate the type I error rates of the proposed method, we simulate the test statistic ***Z*** from a multivariate normal distribution *N*(***0***,***R***), where ***R*** denotes the corresponding LD matrix of the gene *EPB41* (erythrocyte membrane protein band 4.1), which is used in our simulation studies. Authors in [14] reported that gene *EPB*41 colocalizes with *AMPA* receptors at excitatory synapses and mediates the interaction of the *AMPA* receptors with the cytoskeleton. Existing study [15] has demonstrated brain region- and subunit-specific abnormalities in the expression of subunits of the *AMPA* subtype of glutamate receptors in schizophrenia. We consider four different significance levels: *α*=10^−3^,10^−4^,10^−5^, and 2.80×10^−6^. In the simulation studies, p-values of our method and aSPU are estimated by performing 10^6^ times permutations and type I error rates are calculated based on 10^6^ replicates. Table 1 shows the estimated type I error rates. From this table, we can see that the type I error rates of all of the methods are controlled well, which indicate that all the tests are valid.

**Table 1:** Estimated type I error rates for different test methods

*α*-level	ST	S2T	AT	GATES	aSPU	TS
1×10^−3^	1.02×10^−3^	0.97×10^−3^	1.04×10^−3^	1.05×10^−3^	1.00×10^−3^	1.00×10^−3^
1×10^−4^	1.09×10^−4^	0.91×10^−4^	1.06×10^−4^	0.99×10^−4^	1.02×10^−4^	1.00×10^−4^
1×10^−5^	1.10×10^−5^	0.95×10^−5^	1.00×10^−5^	0.90×10^−5^	1.10×10^−5^	1.00×10^−5^
2.8×10^−6^	4.00×10^−6^	3.00×10^−6^	2.50×10^−6^	2.00×10^−6^	3.00×10^−6^	3.00×10^−6^

#### Power comparison

Using the same gene *EPB41*, we conducted extensive simulation studies to assess the power of the proposed method. We simulate 10^4^ summary statistics from *N*(***A***×△,***R***) where ***A*** denotes the signs of associations which are determined by the risk or protective effects of causal variants. △ denotes different settings and determines the effect sizes of causal variants. ***R*** is the corresponding LD matrix of the gene *EPB*41. We simulated three causal SNPs. The effects of the three causal SNPs are deternmined by randomly selecting three numbers either 1 or -1 for ***A***. Then we set the effects of the rest of SNPs in the gene as 0. Table 2 shows the estimated power at 2.80×10^−6^ significance level under three combinations of ***A*** for three settings of △: a set of fixed values of △ and two randomly simulated △, one from uniform distribution, and the other from normal distribution. The proposed TS performs robustly across all scenarios and has the overall best performance compared to the other five test methods. As our proposed method TS belongs to quadratic tests and uses a truncated method to find the true contribution of genetic variants to the association, our method is robust to the direction of effects and weak effect sizes. Among the five comparable tests, even though S2T and GATES methods are unaffected by the directions of causal SNPs, these two methods will suffer a loss of power when the effect sizes are weak, which is indicated by the results of the first three settings (three different fixed values of △). Comparing the results of the last three settings from the same normal distribution of △, the burden test ST has the worst performance when there are differing directions of effects. Because the two adaptive methods, AT and aSPU, are considered to be a combination of the burden test and the quadratic test, these two methods will still be more or less affected by directions and other noises, despite their adaptive nature. The results of the first three settings from fixed effect size of △, and middle three settings from uniform distribution of △, verify this conclusion. TS maintains its power in all of the scenarios, indicating that our proposed test TS is robust to different directions of effects and weak effect sizes.

**Table 2:** Estimated power (%) under 2.8×10^−6^ significance level for different tests. Data are simulated from *N*(***A***×△,***R***). ***A*** has 3 nonzero elements with different signs

nonzero △	nonzero ***A***	AT	S2T	ST	GATES	aSPU	TS
(4,2,1)	(1,1,1)	68.0	8.5	68.5	16.5	83.5	95.5
(4,4,2)	(1,1,-1)	69.0	45.0	45.0	28.5	65.6	93.8
(2,5,4)	(1,-1,-1)	92.5	74.5	76.0	65.5	62.0	98.2
U(1,5)	(1,1,1)	86.0	37.0	87.0	24.0	86.0	97.8
U(2,6)	(1,1,-1)	71.5	71.5	19.0	58.0	68.0	92.0
U(2,6)	(1,-1,-1)	70.0	70.5	19.5	65.5	64.5	92.5
N(3,4)	(1,1,1)	83.0	60.0	83.5	74.5	88.5	95.5
N(3,4)	(1,1,-1)	68.0	70.0	33.0	72.5	69.5	85.7
N(3,4)	(1,-1,-1)	66.0	61.5	33.0	78.0	73.5	87.1

### Application to schizophrenia gWAS summary data set

We applied our method to a schizophrenia (SCZ) GWAS summary data, which was downloaded from the PGC website (see URL https://www.med.unc.edu/pgc/). The GWAS summary data was generated from a meta analysis with 36,989 cases and 113,075 controls, denoted as SCZ [16]. The GWAS summary data consists of the MAF, effect size estimate, odds ratio, and p-value for each SNP contained in a gene. We treat all SNPs that are located in or near a gene as a set to be analyzed for joint association and group all SNPs from 20 kb upstream of a gene to 20 kb downstream of a gene following [2].

In order to better illustrate our TS method and make fair comparisons, we first performed LD pruning for each SNP set by removing those SNPs that have pairwise LD *r*^2^>0.5 with other SNPs. We then removed genome-wide significant SNPs with p-values less than 5×10^−8^ and filtered out those SNPs with MAF <0.05. We set our genome-wide SNP set significance level as 2.8×10^−6^, which is the Bonferroni corrected significance level for the total number of tested SNP sets (17,415 genes). P-values of our method TS and aSPU are estimated by performing 10^7^ permutations, respectively.

**Fig. 1:**
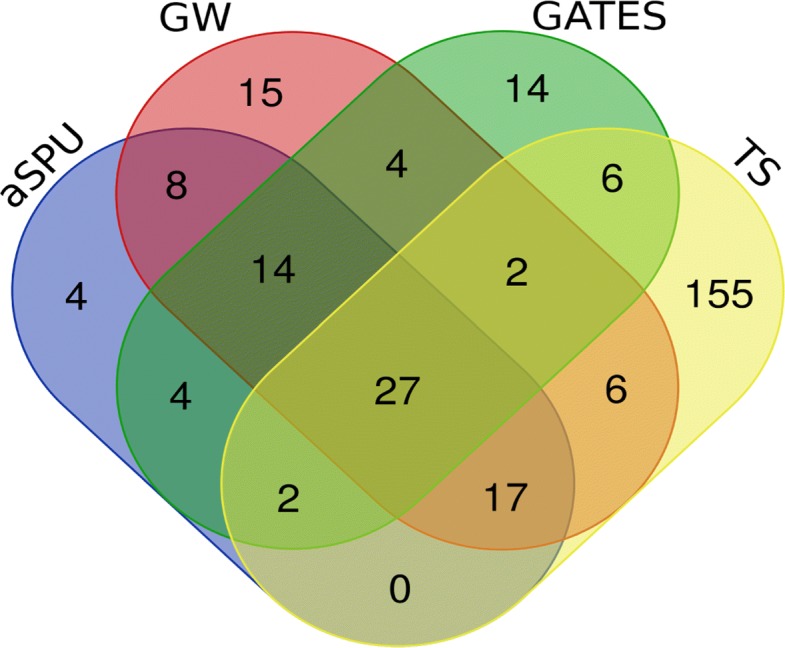
Venn diagram of the number of significant genes identified by TS, aSPU, GATES, and GW for SCZ

We applied our method and the other comparable methods to the SCZ data of 150,064 individuals to identify SCZ-associated genes, then used genome-wide significant SNPs around these genes in the SCZ dataset as partial validation. Figure 1 shows the Venn diagram comparing the number of significant genes identified by our proposed method with the other comparable methods, aSPU, GATES, and Guo and Wu’s method-GW, which represents a super method where we aggregate the identified genes by S2T, ST, and AT. Specifically, TS identified 215 significant genes, aSPU identified 76 significant genes, GATES identified 73 genes, and GW identified 93 significant genes in total (Table 3). Among these 215 significant genes identified by TS, 43 genes (in total 50 unique genes containing significant SNPs in GWAS) contain the genome-wide significant SNPs (p-value <5×10^−8^) within 20 kb in the SCZ data, offering a significant validation of the identified gene. Clearly, our method identified more associated genes than the other three comparison methods.

**Table 3:** Comparison of the four methods using the PGC SCZ and UKB T2D GWAS summary data

Methods	aSPU	GATES	GW	TS
		PGC SCZ		
Total significant genes (m,%)	76 (31, 40.8%)	73 (23, 31.5%)	93 (32, 34.4%)	215 (43, 20%)
Unique significant genes (u,%)	4 (1, 25%)	14 (0, 0%)	15 (1, 6.7%)	155 (13, 8.4%)
		UKB T2D		
Total significant genes (m,%)	54 (25, 46.3%)	40 (12, 30%)	57 (27, 47.4%)	217 (47, 21.7%)
Unique significant genes (u,%)	0 (0, 0%)	7 (1, 14.3%)	1 (0, 0%)	155 (17, 11%)

Note: m denotes the number of significant SNPs in GWAS and u denotes the number of significant SNPs in GWAS. GW denotes a combination of ST, S2T, and AT.

Our method performs better than other methods in terms of the number of significant genes identified. However, each test identified some unique genes missed by the other methods, highlighting that different tests may perform better in different scenarios. Overall, a total of 278 significant and unique genes were identified in the SCZ data across all tests. Supplementary Table S1 shows the significant genes, with associated p-value and minimum p-value, identified by TS, aSPU, GATES, or GW in SCZ, respectively.

Many of these significantly identified genes may have important mechanisms relevant to schizophrenia. Their biological implications in the etiology of schizophrenia are discussed as follows.

miRNA plays a role in gene expression regulation, thus the effect on miRNA expression naturally affects the expression of its target gene(s). Particularly, the presence of altered miRNA expression in brain and peripheral tissues has been implicated in the development of schizophrenia and other psychiatric disorders such as bipolar disorder (BD) and major depression (MD) [17–19]. Several miRNA encoding genes were identified as significant by all four methods (TS, aSPU, GATES, and GW), such as *MIR4677*, *MIR6511A4*, *MIR4267*, *MIR4436B1*, and *MIR137HG*. *MIR137HG* is of particular interest as it is instrumental in neurodevelopment and neuroplasticity [20]. Modulated expression of *MIR137HG* and *MIR137* has been specifically shown to reduce grey matter content in key areas in the brain, which is characteristic of schizophrenia [21, 22]. However, TS identified several miRNA encoding genes that were missed by the other methods, such as *MIR4256*, *MIR6756*, *MIR3652*, *MIR4700*, and *MIR624*. Due to the growing evidence regarding miRNA involvement in psychiatric disorders, some of these genes may be relevant to schizophrenia etiology, and thus may warrant further research.

Voltage-dependent calcium ion channel dysfunction has a long history of being a plausible mechanism for schizophrenic pathology [23]. *CACNA1C* codes for a calcium ion channel subunit, and has been reported to be a target of *miR-137* [19]. Interestingly, TS uniquely identified another calcium ion channel subunit encoding gene, *CACNA2D3*. Due to its similarity to *CACNA1C* and its high expression in the brain [24], *CACNA2D3* may also prove to be a potent factor in schizophrenic development, despite not containing any genome-wide significant SNPs (most significant SNP p = 0.000586), which highlights our method’s robustness to weak effects.

Finally, TS uniquely identified *GRM7* as significant. Glutamate receptor dysfunction has been long studied for its role in schizophrenia development [25, 26], and *GRM7* in particular was recently investigated for its potential as a biomarker for risperidone response [27]. *GRM7* was also previously identified by another proposed method, OWC [7], indicating TS is equivalently adapted for identifying weakly associated signals.

### Application to t2D gWAS summary data sets

We also conducted a comprehensive analysis of fasting glucose GWAS summary data for type 2 diabetes (T2D) from the UK Biobank component of the European DIAMANTE study (denoted as UKB), which included over 440,000 individuals of European ancestry with 19,119 cases and 423,698 controls. The analysis for the dataset was restricted to HRC variants and was conducted using the UK Biobank Resource under Application Number 9161 (McCarthy). The GWAS summary data can be downloaded from http://www.type2diabetesgenetics.org/informational/data. Similar to the SCZ dataset, the UKB summary data also consists of MAF, effect size estimate, odds ratio, and p-value for each SNP in genes. We applied the same procedure used to filter and analyze the SCZ data on the UKB data. We used 0.05/17,495≈2.8×10^−6^ as the significance level and performed 10^7^ permutations for our proposed method TS and aSPU, respectively.

**Fig. 2:**
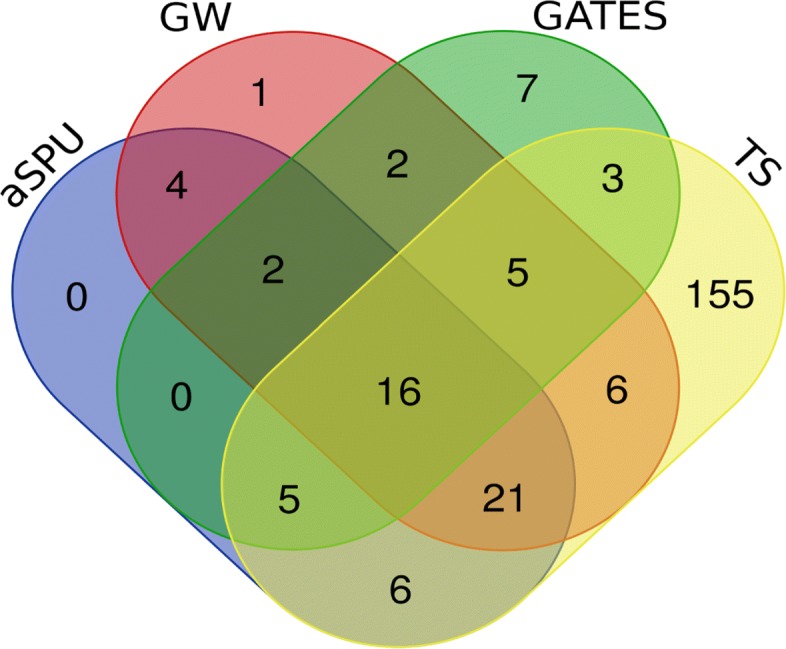
Venn diagram of the number of significant genes identified by TS, aSPU, GATES, and GW for UKB

Figure 2 shows the Venn diagram comparing the number of significant genes identified by our proposed method compared with aSPU, GATES and GW. TS identified 217 significant genes, whereas aSPU, GATES, and GW identified 54, 40, and 57 significant genes, respectively (Table 3). Among these 217 significant genes identified by TS, 47 genes contain the genome-wide significant SNPs (p-value <5×10^−8^) within 20 kb in the UKB data. In total 78 genes in the T2D GWAS contain significant SNPs. That is, our TS method verified 60.26% of the entire genes containing significant SNPs. Based on the UKB analysis, we can further conclude that our TS method performed the best compared to the other methods in terms of the number of significant genes identified. The 233 significant and unique genes identified by all of the four methods are provided in Supplementary Table S2.

In the UKB dataset, *ADIPOR2* was identified as significant by all methods except GW, which was just below significance (p = 2.6E −5). Adiponectin is an adipokine involved in metabolic control, and has insulin sensitizing effects. Expression of adiponectin is found to be down-regulated as insulin resistance is developing, and is also associated with anti-inflammatory and other protective effects [28]. *ADIPOR2* codes for an adiponectin receptor, dysfunction of which affects adiponectin’s ability to exert its regulatory effects. Deletion of the gene entirely in mice models demonstrates ineffective *ADIPOR2* function in development of type 2 diabetes [29], and recovery of receptor function has been considered for a potential target in preventing diabetic nephropathy in mice [30]. Also, variations in *ADIPOR2* have previously been demonstrated to be associated with type 2 diabetes [31, 32]. Bountiful evidence of the effects of adiponectin and *ADIPOR2* expression on T2D pathology serves to further highlight the validity of the truncated statistic method. T2D pathogenesis has an inflammatory component, which connects the inability of pancreatic beta cells to maintain sufficient insulin levels in the presence of developing resistance with various cellular stresses that either induces or is associated with an inflammatory respons [33]. In particular, activation of the JNK pathway is associated with a reduction in insulin gene expression and subsequent development of T2D [34]. Our method identified *MAP4K5* as significant (most significant SNP p = 1.7E-8), which was missed by the other three methods. *MAP4K5* encodes for a component in mitogen-activated protein kinase (MAPK) cascade, a signaling pathway which mediates activation of the JNK pathway via tumour necrosis factor *α* (TNF- *α*) [35, 36]. Additionally, a particular study identified key protective polymorphisms in *MAP4K5* which may affect JNK activation and thus T2D development [37].

We further performed a verfication study for T2D using an independent T2D GWAS summary data obtained from the DIAbetes Genetics Replication And Meta-analysis (DIAGRAM) Consortium (http://diagram-consortium.org/downloads.html). The stage 1 analyses of DIAGRAM comprised a total of 26,676 T2D cases and 132,532 control participants from 18 GWAS. In stage 1, in each study, logistic regression association analysis of T2D was performed with genotype dosage using an additive genetic model including covariates age, sex and principal components derived from the genetic data to account for population stratification. In fifteen of the repeated studies, researchers also adjusted for body mass index (BMI) [38]. The GWAS summary data consists of the MAF, effect size estimate, odds ratio, and p-value for each SNP contained in a gene. We applied the proposed TS method and five other methods to the DIAGRAM stage 1 GWAS summary data in order to verify T2D associated genes, which were identified using the GWAS summary data from the European DIAMANTE study (UKB). Table 4 shows that among 217 genes identified in UKB with the TS method, 32 genes were verified in the DIAGRAM data. The verification rate is 14.7%, which is greater than 8.7% of the GW method (S2T, ST, and AT combined) and 9.2% of the aSPU method.

**Table 4:** Verification study for UKB T2D using GWAS summary data obtained from DIAGRAM

Methods	Number of significant genes from UKB	Verified genes from Diagram	Verfied percentage
GW	57	5	8.7%
aSPU	54	5	9.2%
GATES	40	8	20.0%
TS	217	32	14.7%

Note: GW denotes a combination of ST, S2T, and AT.

We analyzed genes uniquely identified by a method and showed how many of them included the significant SNPs in GWAS in Table 3. In the PGC SCZ data, the proposed TS method (8.4%) performs better than the GW (6.7%) and GATES (0%). Although the ratio 25% of sSPU is greater then 8.4% of TS, aSPU uniquely identified 4 genes and only 1 of them included the significate SNPs in GWAS. In the UKB T2D data, the proposed TS method (11%) performs better than the GW (0%) and aSPU (0%). The GATES uniquely identified 7 T2D associated genes, and only 1 of them included the significant SNPs in GWAS.

In summary, in both the SCZ and UKB summary data-sets, several genes which contain genome-wide significant SNPs were identified by all of the methods considered, including TS, further highlighting the validity of our proposed method. Additionally, TS identified genes in both datasets that were missed by the other methods, which demonstrates its ability to maintain power across a range of scenarios as well as overcome the limitations discussed previously.

## Discussion

In this paper, we propose a novel gene-based genetic association test, the truncated statistic method (TS), where we use a truncated test to find the estimated contribution of genetic variants based on a quadratic statistic. The TS method can overcome the shortcomings of both burden tests and quadratic tests: different directions of effects for burden tests and the large number of other noises for quadratic tests. When our data satisfies some special conditions (see Appendix A), our method can reduce to the score test. If we focus on the summary data obtained from rare variants analysis, we can set the weight on each Z statistic of each SNP that has the beta distribution density function with pre-specified shape parameters 1 and 25 being in row evaluated at the corresponding sample MAF in the data, similar to the method of SKAT. Through simulation studies and real data analyses, we demonstrate that the proposed test TS, often outperforms other comparison methods such as ST, S2T, AT, aSPU, and GATES, which are some of the most popular methods based on summary data.

When we perform association tests based on a large number of genes in whole-genome sequencing studies, different disease models may exist: some of the models are likely to include many causal variants whose effects are in the same directions while other models may include few causal variants or causal variants whose effects are in different directions; or some of the models are likely to include many weakly associated variants while other models may include a few strongly associated variants. Because the true disease model is usually unknown, there is no uniformly most powerful test to detect single trait associated genes; an association test may perform well for one dataset, but not necessarily for another. For example, both schizophrenia and type 2 diabetes are representative of complex diseases with common but often weakly associated variants, in which many of these variants may be working in tandem to produce the disease. A robust, flexible method such as TS is needed to better elucidate these weakly associated variants so that their role in disease etiology can be understood further. Thus, the proposed TS can be an attractive tool for many situations, because it adapts to the underlying biological disease model by selecting the true contribution of genetic variants.

Our proposed method provides an alternative approach with extreme scalability and robust performance. This method only needs the publicly available GWAS summary statistics as input, without the need to access raw genotype and phenotype data. TS incorporates the inverse of the LD matrix. Thus, it can account for the LD information among SNPs. In order to guarantee that the LD matrix is invertible in real data analysis, we suggest to perform SNP pruning first [39] before applying TS in the GWAS summary data. We expect that researchers will be able to identify novel disease associated genes by employing the proposed method to analyze publicly available GWAS summary data and shed more light onto the underlying mechanisms of diseases. In our paper, our proposed method mainly focused on single trait GWAS summary data. However, it can be easily extended to multiple traits GWAS summary data. We have implemented the proposed method in an C code, which is publicly available online at github https://github.com/Jianjun-CN/c-code-for-TS.

## Conclusions

We proposed a powerful gene based test TS. Simulation studies and real data analyses demontrated that TS outperformed existing methods. It can be employed to detect novel associated genes using GWAS summary data.

## Method

Suppose we have GWAS summary data including MAF, estimated effect size, standard deviation, p-value, test statistic, for each SNP from a GWAS study. We aim to propose a novel gene based association test using the GWAS summary data. Assume *M* genetic variants in a considered region (a gene or a pathway) are associated with a phenotypic trait with effect sizes ***β***=(*β*_1_,…,*β*_*M*_). Denote ***Z***=(*Z*_1_,…,*Z*_*M*_) as a vector of test statistics used to test the genetic associations for the *M* genetic variants. The null hypothesis is *H*_0_:***β***=0 and the alternative hypothesis is *H*_1_:***β***≠0, which means that at least one of the elements of ***β*** does not equal to zero. For each pair of Z statistics, under the null hypothesis, we have:

1 $$ Cov(Z_{k},Z_{l})=r_{kl}  $$

where *r*_*kl*_ denotes the LD between the *k*^*th*^ SNP and the *l*^*th*^ SNP [8]. We can assume that the p-values are calculated based on the Z-statistic, even though the p-values may be computed based on non-Z statistics. This assumption will not affect our conclusion about the proposed test. For p-values obtained from non-Z statistics (such as chi-square test, or t-test), we can transform the corresponding p-values into Z statistics by using *Z*_*m*_=sign(*β*_*m*_)*Ψ*^−1^(1−*p*_*m*_/2) for the *m*^*th*^ SNP when we know the p-value, where *Ψ*(·) is the cumulative distribution function (CDF) of a standard normal distribution and *β*_*m*_ denotes the corresponding estimated effect size contained in the GWAS summary results.

It is reasonable to assume that ***Z*** follows a multivariate normal distribution with mean ***0*** and correlation matrix ***R*** under the null hypothesis, where ***R*** denotes the estimated null correlation matrix computed based on the variant linkage disequilibrium (LD). We propose a test statistic that includes only Z statistics with a true contribution to the association of a genetic variant under the alternative hypothesis. Here, we adopt the truncated statistic method for combining statistical evidence, which has been suggested for such an analysis in literature [40, 41]. For a given *τ*>0, we let ***Z***(*τ*) be the sub-vector of ***Z*** satisfying |*Z*_*m*_|≥*τ*. That is, only the statistics in the vector ***Z*** with an absolute value greater than or equal to *τ* will be kept. Similarly, we let ***R***(*τ*) be a sub-matrix of ***R*** representing the correlation matrix corresponding to ***Z***(*τ*). Define the test statistic based on ***Z***(*τ*) and ***R***(*τ*) as:

2 $$ T_{\tau}=\boldsymbol{Z}(\tau)^{T}\boldsymbol{R}(\tau)^{-1}\boldsymbol{Z}(\tau)  $$

When *τ* is large, *T*_*τ*_ can be undefined if |*Z*_*m*_|<*τ* for all *m*. In this case, we define *T*_*τ*_=0. Then, our test statistic is:

3 $$ S_{T}=\max_{\tau>0}T_{\tau}  $$

Usually, we select the value *τ* from the second minimum value of ***Z*** to the maximum value of ***Z***, because we have proven that the statistic *T*_*τ*_ containing more than one Z-statistic is greater than the statistic *T*_*τ*_ that contains only one Z-statistic (see Appendix A). That is, we only need to compute *T*_*τ*_ at most *M*−1 times to get the maximum value of *S*_*T*_. The asymptotic distribution of *S*_*T*_ does not follow a standard distribution but can be evaluated by permutation methods.

We use the following steps to evaluate the distribution of *S*_*T*_ under the null hypothesis:

1. Suppose we permute B times. For the *b*^*th*^ permutation, we obtain the Z statistics, ***Z***^*b*^, generated from the multivariate normal distribution *N*(***0***,***R***) where *b*=0 represents the original Z statistics.
2. Scan all possible *τ* values, correspondingly we search from the second minimum value of ***Z***^*b*^ to the maximum value of ***Z***^*b*^, then, we get the test statistic $S_{T}^{(b)}$ for the *b*^*th*^ step.
3. Repeat B times permutations and then the p-value of *S*_*T*_ is given by:

$$P_{T}=\frac{\#\{b:S_{T}^{(b)}>S_{T}^{(0)}, \ b=1,\cdots,B\}}{B}$$

### Comparison of methods

We evaluate the performance of the proposed method (TS) by comparing it with the five aforementioned methods: 1) adaptive sum of powered score tests (aSPU) [10]; 2) gene-based association test that uses extended Simes procedure (GATES) [9]; 3) three methods proposed by [11]: sum test (ST), squared sum test (S2T), and adaptive test (AT).

Please find the brief introduction about these methods and their notations as follows:

1. Sum test (ST), $B=\sum _{m=1}^{M}Z_{m}$, a type of burden test statistic [3].
2. Squared sum test (S2T), $Q=\sum _{m=1}^{M}Z_{m}^{2}$, a type of SKAT statistic [4] and equivalent to the weighted sum of squared score (SSU) test [42].
3. Adaptive test (AT), *T*= min*ρ*∈[0,1]*P*(*Q*_*ρ*_), where *Q*_*ρ*_=(1−*ρ*)*Q*+*ρB*^2^ and *P*(*Q*_*ρ*_) denotes the corresponding p-value.
4. Adaptive sum of powered score tests (aSPU), aSPUs= min*γ*∈*ΓP*_*SPUs*(*γ*)_ where $SPUs(\gamma)=\sum _{m=1}^{M}Z_{m}^{\gamma }$.
5. Gene-based association test that uses extended Simes procedure (GATES), $p_{GATES}=\min \big (\frac {m_{e}p_{(j)}}{m_{e(j)}}\big)$ where *m*_*e*_ is the effective number of independent p-values among the M SNPs, *p*_(*j*)_ is the *j*^*th*^ smallest p-value and *m*_*e*(*j*)_ is the effective number of independent p-values among the top *j* SNPs.

Because ***Z***∼MVN(***0***,***R***), B=$1_{M}^{'}\boldsymbol {Z}\sim N(0,1_{M}^{'}\boldsymbol {R}1_{M})$, where 1_*M*_ denotes a column vector of length *M* where every element is equal to one. Therefore, $\frac {B^{2}}{1_{M}^{'}\boldsymbol {R}1_{M}}$ follows the *χ*_1_ distribution. Under the null hypothesis, the statistic of the squared sum test Q=$\phantom {\dot {i}\!}\boldsymbol {Z}^{'}\boldsymbol {Z}$ has an asymptotic distribution as the weighted sum of independent *χ*_1_ with weights being the eigenvalues of ***R***. We compute the p-value of the *T* statistic of the adaptive test using an one-dimensional numerical integration, where we will search over *ρ*∈(0,0.01,0.04,0.09,0.16,0.25,0.5,1) following [43]. We can estimate the p-values of the three statistics proposed by [11] using the “sats" function in the “mkatr" package in R. We estimate the p-values of the adaptive sum of powered score tests (aSPU) using the Monte Carlo simulations, which can be obtained using the “aSPUs” function in the “aSPU” package in R. We use the “gates” function in the “COMBAT" package in R to calculate the p-value of the gene-based association test that uses extended Simes procedure (GATES).

## Appendix

Assume that ***Z***=(*Z*_1_,⋯,*Z*_*M*_) represents a vector of Z statistics following a multivariate normal distribution with mean ***0*** and covariance matrix ***R***, where ***R*** is the LD matrix between SNPs. When we try to adapt the truncated statistic method for combining statistical evidence, we want to know whether the test statistic in equation (2) will increase or not when we add a new Z statistic.

If *τ* changes from a large value to a small value, the number of Z statistics added in the test statistic will increase as *τ* changes. Suppose we have selected *n* Z statistics, ***Z***_1_=(*Z*_1_,⋯,*Z*_*n*_)^*T*^ and have a corresponding covariance matrix *Σ*_1_ for *n*<*M*. We can then write the test statistic as $\boldsymbol {Z}_{1}^{T}\Sigma _{1}^{-1}\boldsymbol {Z}_{1}$. Suppose we add one Z statistic, *Z*_*n*+1_, to the test statistic, when *τ* gets smaller. The corresponding test Z statistic can be written as:

4 $$ (\boldsymbol{Z}_{1}^{T}, Z_{n+1}) \left(\begin{array}{ll} \Sigma_{1} & B \\ B^{T} & 1 \end{array} \right)^{-1} \left(\begin{array}{ll} \boldsymbol{Z}_{1} \\ Z_{n+1} \end{array} \right)  $$

where B represents the correlation between the *Z*_*n*+1_ statistic with the first *n* Z statistics. From the theory of block matrix inversion, we have:

5 $$ \left(\begin{aligned} \Sigma_{1} & B \\ B^{T} & 1 \end{aligned} \right)^{-1} = \left(\begin{aligned} \Sigma_{1}^{-1}+\Sigma_{1}^{-1}BB^{T}\Sigma_{1}^{-1}(1-B^{T}\Sigma_{1}^{-1}B)^{-1} &\quad -\Sigma_{1}^{-1}B(1-B^{T}\Sigma_{1}^{-1}B)^{-1} \\ -(1-B^{T}\Sigma_{1}^{-1}B)^{-1}B^{T}\Sigma_{1}^{-1} &\quad\quad(1-B^{T}\Sigma_{1}^{-1}B)^{-1} \end{aligned} \right)  $$

Because $(1-B^{T}\Sigma _{1}^{-1}B)^{-1}$ is a constant (not a matrix), we define $C=(1-B^{T}\Sigma _{1}^{-1}B)^{-1}$ and rewrite the Eq. 5 as:

$$\begin{array}{*{20}l} \left(\begin{array}{ll} \Sigma_{1} & B \\ B^{T} & 1 \end{array} \right)^{-1} &= \left(\begin{array}{ll} \Sigma_{1}^{-1}+\Sigma_{1}^{-1}BB^{T}\Sigma_{1}^{-1}*C & -\Sigma_{1}^{-1}B*C \\ -C*B^{T}\Sigma_{1}^{-1} & C \end{array} \right)\\ &=C \left(\begin{array}{ll} \frac{1}{C}\Sigma_{1}^{-1}+\Sigma_{1}^{-1}BB^{T}\Sigma_{1}^{-1} & -\Sigma_{1}^{-1}B \\ -B^{T}\Sigma_{1}^{-1} & 1 \end{array} \right) \end{array} $$

Then, the truncated statistic (4) is equivalent to:

$$\begin{array}{*{20}l} &(\boldsymbol{Z}_{1}^{T}, Z_{n+1}) \left(\begin{array}{ll} \Sigma_{1} & B \\ B^{T} & 1 \end{array} \right)^{-1} \left(\begin{array}{l} \boldsymbol{Z}_{1} \\ Z_{n+1} \end{array} \right)\\ =&C (\boldsymbol{Z}_{1}^{T}, Z_{n+1}) \left(\begin{array}{ll} \frac{1}{C}\Sigma_{1}^{-1}+\Sigma_{1}^{-1}BB^{T}\Sigma_{1}^{-1} & -\Sigma_{1}^{-1}B \\ -B^{T}\Sigma_{1}^{-1} & 1 \end{array} \right) \left(\begin{array}{l} \boldsymbol{Z}_{1} \\ Z_{n+1} \end{array} \right)\\ =& \boldsymbol{Z}_{1}^{T}\Sigma_{1}^{-1}\boldsymbol{Z}_{1}+C\Big[ (\boldsymbol{Z}_{1}^{T}\Sigma_{1}^{-1}B)^{2}-2Z_{n+1}\boldsymbol{Z}_{1}^{T}\Sigma_{1}^{-1}B+Z_{n+1}^{2} \Big]\\ =&\boldsymbol{Z}_{1}^{T}\Sigma_{1}^{-1}\boldsymbol{Z}_{1}+C\Big(\boldsymbol{Z}_{1}^{T}\Sigma_{1}^{-1}B-Z_{n+1}\Big)^{2} \end{array} $$

If we want to know whether the test statistic will increase or not when we add a new Z statistic, we only need to check whether the condition $C=(1-B^{T}\Sigma _{1}^{-1}B)^{-1}$ is greater than zero or not. It means that when $B^{T}\Sigma _{1}^{-1}B<1$, the truncated statistics will increase, and will not increase otherwise. For the special case, *n*=1, we always have $B^{T}\Sigma _{1}^{-1}B<1$. It means the Z statistics contained in the truncated statistic will always be greater than 1. This is also in line with our thought, as we view our proposed method as a complementary approach to single trait single variant association tests. When these *n*+1 Z statistics are all independent, it means *B*=0. Thus, the truncated statistic degenerates to a summation of the square of these *n*+1 Z statistics, and our method becomes equivalent to the score test.

## Supplementary information

**Additional file 1** The TS and the other comparison methods are applied to the SCZ data.

**Additional file 2** The TS and the other comparison methods are applied to the UKB data.

## Data Availability

The datasets used and/or analysed during the current study are available from the corresponding author on reasonable request. The proposed methods are implemented in an C code available at https://github.com/Jianjun-CN/c-code-for-TS The original SCZ GWAS summary data can be downloaded at the PGC site https://www.med.unc.edu/pgc/. The original UKB GWAS summary data can be downloaded at http://www.type2diabetesgenetics.org/informational/data.

## Abbreviations

GWASs: Genome-wide association studies
TS: Truncated statistic method
T2D: Type 2 diabetes
MAF: Minor allele frequency
SNPs: Single nucleotide polymorphisms
WSS: Weighted sum statistic
SKAT: Sequence kernel association test
SSU: Sum of squared score test
GATES: Gene-based association test that uses extended Simes procedure
GPA: Gene-based p-value adaptive combination approach
aSPU: Adaptive sum of powered score tests
ST: Sum test
S2T: Squared sum test
AT: Adaptive test
SPU: Sum of powered score
PGC: Psychiatric Genomics Consortium
LD: Linkage disequilibrium
SCZ: Schizophrenia
UKB: UK Biobank component of the European DIAMANTE study

## References

[1] Manolio TA, Collins FS, Cox NJ, Goldstein DB, Hindorff LA, Hunter DJ, Cho JH (2009). Finding the missing heritability of complex diseases. Nature.

[2] Wu MC, Kraft P, Epstein MP, Taylor DM, Chanock SJ, Hunter DJ, Lin X (2010). Powerful SNP-set analysis for case-control genome-wide association studies. Am J Hum Genet.

[3] Madsen BE, Browning SR (2009). A groupwise association test for rare mutations using a weighted sum statistic. PLoS Genet.

[4] Wu MC, Lee S, Cai T, Li Y, Boehnke M, Lin X (2011). Rare-variant association testing for sequencing data with the sequence kernel association test. Am J Hum Genet.

[5] Pan W (2011). Relationship between genomic distance-based regression and kernel machine regression for multi-marker association testing. Genet Epidemiol.

[6] Lee S, Wu MC, Lin X (2012). Optimal tests for rare variant effects in sequencing association studies. Biostatistics.

[7] Zhang J, Gonzales S, Liu J, Wang X. An optimally weighted combination method to detect novel disease associated genes using publicly available GWAS summary data. bioRxiv 709808. 2019. 10.1186/s12859-020-3511-0 PMC7199321 32366212

[8] Zhang J, Zhao Z, Guo X, Guo B, Wu B. Powerful statistical method to detect disease associated genes using publicly available GWAS summary data. bioRxiv 478321. 2018. 10.1002/gepi.22251 31392781

[9] Li MX, Gui HS, Kwan JS, Sham PC (2011). GATES: a rapid and powerful gene-based association test using extended Simes procedure. Am J Hum Genet.

[10] Kwak IY, Pan W (2015). Adaptive gene-and pathway-trait association testing with GWAS summary statistics. Bioinformatics.

[11] Guo B, Wu B (2018). Statistical methods to detect novel genetic variants using publicly available GWAS summary data. Comput Biol Chem.

[12] Guo B, Wu B (2018). Powerful and efficient SNP-set association tests across multiple phenotypes using GWAS summary data. Bioinformatics.

[13] 1000 Genomes Project Consortium (2012). An integrated map of genetic variation from 1,092 human genomes. Nature.

[14] Shen L, Liang F, Walensky LD, Huganir RL (2000). Regulation of AMPA receptor GluR1 subunit surface expression by a 4.1 N-linked actin cytoskeletal association. J Neurosci.

[15] Tucholski J, Simmons MS, Pinner AL, McMillan LD, Haroutunian V, Meador-Woodruff JH (2013). N-linked glycosylation of cortical NMDA and kainate receptor subunits in schizophrenia. Neuroreport.

[16] Schizophrenia Working Group of the Psychiatric Genomics Consortium (2014). Biological insights from 108 schizophrenia-associated genetic loci. Nature.

[17] Maffioletti E, Tardito D, Gennarelli M, Bocchio Chiavetto L. Micro spies from the brain to the periphery: New clues from studies on microRNAs in neuropsychiatric disorders. Front Cell Neurosci. 2014; 8. 10.3389/fncel.2014.00075. 10.3389/fncel.2014.00075 PMC3949217 24653674

[18] Duan J, Shi J, Fiorentino A, Leites C, Chen X, Moy W, Gejman PV (2014). A Rare Functional Noncoding Variant at the GWAS-Implicated MIR137/MIR2682 Locus Might Confer Risk to Schizophrenia and Bipolar Disorder. Am J Hum Genet.

[19] Kwon E, Wang W, Tsai L-H (2013). Validation of schizophrenia-associated genes CSMD1, C10orf26, CACNA1C and TCF4 as miR-137 targets. Mol Psychiatry.

[20] Mahmoudi E, Cairns MJ (2017). MiR-137: An important player in neural development and neoplastic transformation. Mol Psychiatry.

[21] Wright C, Gupta CN, Chen J, Patel V, Calhoun VD, Ehrlich S, Turner JA (2016). Polymorphisms in MIR137HG and microRNA-137-regulated genes influence gray matter structure in schizophrenia. Transl Psychiatry.

[22] Vita A, De Peri L, Deste G, Sacchetti E (2012). Progressive loss of cortical gray matter in schizophrenia: A meta-analysis and meta-regression of longitudinal MRI studies. Transl Psychiatry.

[23] Lidow MS (2003). Calcium signaling dysfunction in schizophrenia: A unifying approach. Brain Res Rev.

[24] Sayers EW, Agarwala R, Bolton EE, Brister JR, Canese K, Clark K, Holmes JB (2019). Database resources of the national center for biotechnology information. Nucleic Acids Res.

[25] Ohtsuki T, Koga M, Ishiguro H, Horiuchi Y, Arai M, Niizato K, Arinami T (2008). A polymorphism of the metabotropic glutamate receptor mGluR7 (GRM7) gene is associated with schizophrenia. Schizophr Res.

[26] Li W, Ju K, Li Z, He K, Chen J, Wang Q, Shi Y (2016). Significant association of GRM7 and GRM8 genes with schizophrenia and major depressive disorder in the Han Chinese population. Eur Neuropsychopharmacol J Eur Coll europsychopharmacol.

[27] Sacchetti E, Magri C, Minelli A, Valsecchi P, Traversa M, Calza S, Gennarelli M (2017). The GRM7 gene, early response to risperidone, and schizophrenia: A genome-wide association study and a confirmatory pharmacogenetic analysis. Pharmacogenomics J.

[28] Lihn AS, Pedersen SB, Richelsen B (2005). Adiponectin: action, regulation and association to insulin sensitivity. Obes Rev.

[29] Liu Y, Michael MD, Kash S, Bensch WR, Monia BP, Murray SF, Reifel-Miller A (2007). Deficiency of Adiponectin Receptor 2 Reduces Diet-Induced Insulin Resistance but Promotes Type 2 Diabetes. Endocrinology.

[30] Park HS, Lim JH, Kim MY, Kim Y, Hong YA, Choi SR, Park CW (2016). Resveratrol increases AdipoR1 and AdipoR2 expression in type 2 diabetic nephropathy. J Transl Med.

[31] Vaxillaire M, Dechaume A, Vasseur-Delannoy V, Lahmidi S, Froguel P (2006). Genetic analysis of ADIPOR1 and ADIPOR2 candidate polymorphisms for type 2 diabetes in the caucasian population. Diabetes.

[32] Damcott CM, Ott SH, Pollin TI, Reinhart LJ, Shuldiner A (2005). Genetic variation in adiponectin receptor 1 and adiponectin receptor 2 is associated with type 2 diabetes in the old order amish. Diabetes.

[33] Donath MY, Shoelson SE (2011). Type 2 diabetes as an inflammatory disease. Nat Rev Immunol.

[34] Kaneto H, Xu G, Fujii N, Kim S, Bonner-Weir S, Weir GC (2002). Involvement of c-Jun N-terminal Kinase in Oxidative Stress-mediated Suppression of Insulin Gene Expression. J Biol Chem.

[35] Shi C-S, Leonardi A, Kyriakis J, Siebenlist U, Kehrl JH (1999). TNF-Mediated Activation of the Stress-Activated Protein Kinase Pathway: TNF Receptor-Associated Factor 2 Recruits and Activates Germinal Center Kinase Related. J Immunol.

[36] Shi C-S, Kehrl JH (2003). Tumor Necrosis Factor (TNF)-induced Germinal Center Kinase-related (GCKR) and Stress-activated Protein Kinase (SAPK) Activation Depends upon the E2/E3 Complex Ubc13-Uev1A/TNF Receptor-associated Factor 2 (TRAF2). J Biol Chem.

[37] Gu Y, Luo T, Yang J, Zhang D, Dai M, Jian W, Luo M (2006). The -822G/A polymorphism in the promoter region of the MAP4K5 gene is associated with reduced risk of type 2 diabetes in Chinese Hans from Shanghai. J Hum Genet.

[38] Scott RA, Scott LJ, Mägi R, Marullo L, Gaulton KJ, Kaakinen M, Jackson AU (2017). An expanded genome-wide association study of type 2 diabetes in Europeans. Diabetes.

[39] Huang H, Chanda P, Alonso A, Bader JS, Arking DE (2011). Gene-based tests of association. PLoS Genet.

[40] Zaykin DV, Zhivotovsky LA, Westfall PH, Weir BS (2002). Truncated product method for combining P-values. Genet Epidemiol.

[41] Li Y, Feng T, Zhu X (2011). Detecting association with rare variants for common diseases using haplotype-based methods. Stat Interface.

[42] Pan W (2009). Asymptotic tests of association with multiple SNPs in linkage disequilibrium. Genet Epidemiol.

[43] Wu B, Guan W, Pankow JS (2016). On efficient and accurate calculation of significance p-values for sequence kernel association testing of variant set. Ann Hum Genet.

